# Supplementary figures and images for: Unmet clinical needs for COVID-19 tests in UK health and social care settings
Source: PLoS One. 2020 Nov 12;15(11):e0242125. doi: 10.1371/journal.pone.0242125 (PMC7660574; doi:10.1371/journal.pone.0242125)

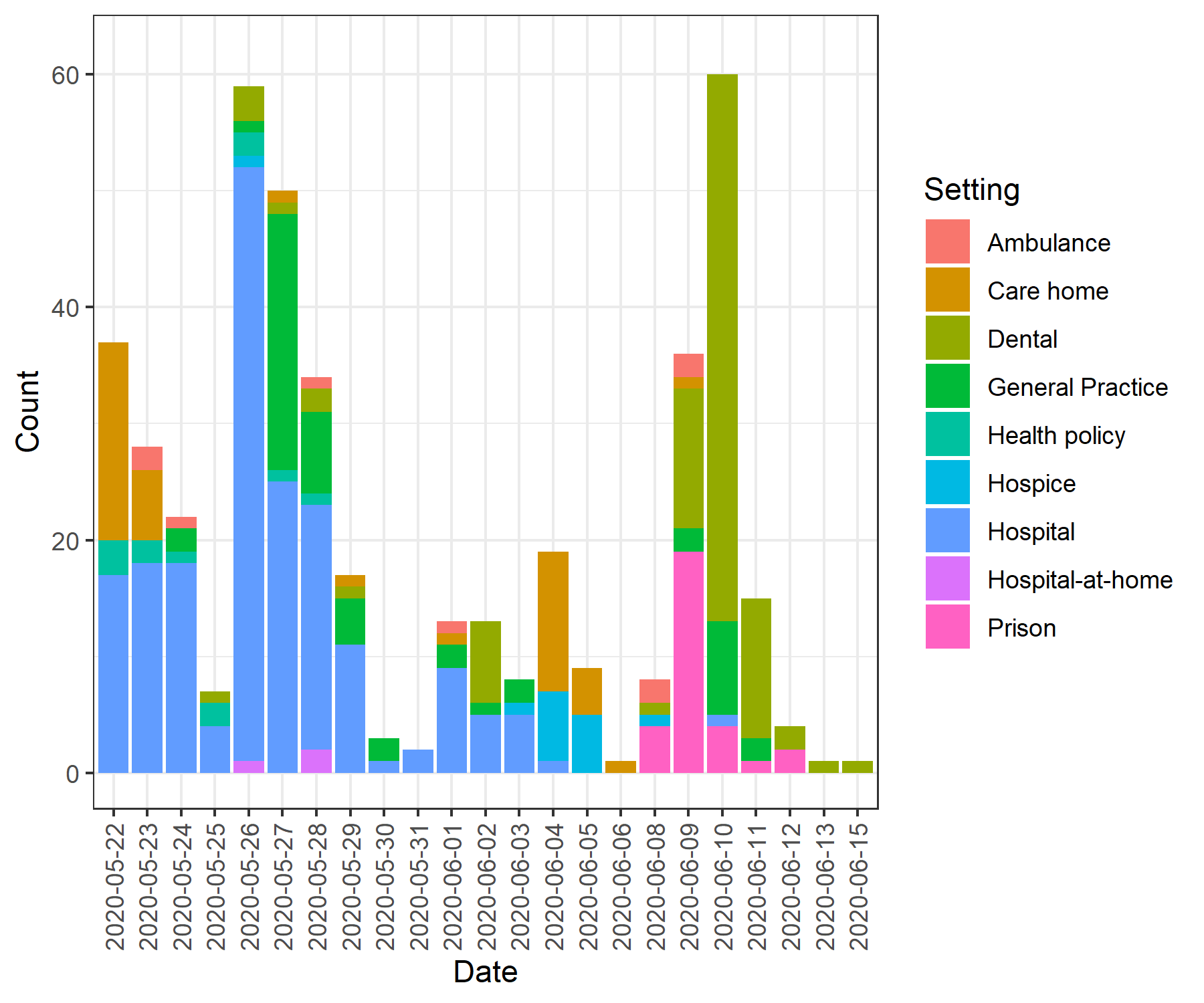

Supplement: S1 Fig — (TIF) [file pone.0242125.s001.tif]
