# Supplementary material for: Unmet clinical needs for COVID-19 tests in UK health and social care settings
Source: PLoS One. 2020 Nov 12;15(11):e0242125. doi: 10.1371/journal.pone.0242125 (PMC7660574; doi:10.1371/journal.pone.0242125)
Supplement: S1 Table — Percentages in parenthesis refer to the total number of respondents for that setting. (DOCX) [file pone.0242125.s002.docx]

**Supporting tables**

S Table 1 – Summary of the qualitative analysis of the new stipulated use cases proposed by the respondents in addition to the list designed by the authors. Percentages in parenthesis refer to the total number of respondents for that setting.

|  | Population | Use cases | **Hospital** | **Care homes** | **General Practice** | **Dental setting** | **Prison** |
| --- | --- | --- | --- | --- | --- | --- | --- |
| **New use cases** | For patients | Screening | 2 | 0 | 0 | 0 | 0 |
|  |  | Diagnostic | 1 | 1 | 0 | 0 | 0 |
|  |  | Prognostic | 0 | 0 | 1 | 0 | 0 |
|  |  | Monitoring | 0 | 0 | 0 | 0 | 0 |
|  | For workers | Screening | 0 | 0 | 0 | 0 | 0 |
|  |  | Diagnostic | 0 | 0 | 0 | 0 | 0 |
|  |  | Prognostic | 0 | 0 | 0 | 0 | 0 |
|  |  | Monitoring | 1 | 0 | 0 | 0 | 0 |
|  | For community | Screening | 1 | 0 | 3 | 0 | 0 |
|  |  | Diagnostic | 1 | 0 | 0 | 0 | 0 |
|  |  | Prognostic | 2 | 0 | 2 | 0 | 0 |
|  |  | Monitoring | 0 | 1 | 0 | 0 | 0 |
|  | **Total (%)** |  | **8 (4%)** | **2 (5%)** | **6 (11%)** | **0** | **0** |
| **Existing use cases** | For patients | Screening | 3 | 1 | 0 | 2 | 0 |
|  |  | Diagnostic | 2 | 0 | 1 | 0 | 0 |
|  |  | Prognostic | 0 | 0 | 2 | 0 | 0 |
|  |  | Monitoring | 0 | 0 | 0 | 0 | 0 |
|  | For workers | Screening | 6 | 3 | 1 | 3 | 1 |
|  |  | Diagnostic | 0 | 0 | 0 | 0 | 0 |
|  |  | Prognostic | 0 | 0 | 0 | 0 | 0 |
|  |  | Monitoring | 3 | 0 | 0 | 0 | 0 |
|  | For community | Screening | 0 | 1 | 2 | 0 | 1 |
|  |  | Diagnostic | 0 | 0 | 0 | 0 | 0 |
|  |  | Prognostic | 0 | 0 | 0 | 0 | 0 |
|  |  | Monitoring | 0 | 0 | 0 | 0 | 0 |
|  | **Total (%)** |  | **14 (7%)** | **5 (11%)** | **6 (11%)** | **5 (5%)** | **2 (7%)** |
| Comments/ incomplete use cases | | | 8 | 7 | 2 | 0 | 1 |
