# Supplementary material for: Unmet clinical needs for COVID-19 tests in UK health and social care settings
Source: PLoS One. 2020 Nov 12;15(11):e0242125. doi: 10.1371/journal.pone.0242125 (PMC7660574; doi:10.1371/journal.pone.0242125)
Supplement: S2 Table — (DOCX) [file pone.0242125.s003.docx]

*S Table 2 – Qualitative analysis of new stipulated use cases proposed by respondents*

| **Hospital** | | | |  |
| --- | --- | --- | --- | --- |
| **N** | **Excerpt** | **Use case** | **Comment/ use case** | **Final Adjudication** |
| 1 | "A test to distinguish infectious (active) infection from recent (recovering) infection." | A test to distinguish infectious infection from recent no more infectious infections | New use case | Diagnostic |
| 2 | "Nosocomial infection is a major threat in hospitals with limited isolation facilities" |  | Comment |  |
| 3 | "All health care professionals " |  | Not enough to extract a use case |  |
| 4 | "Turnaround time and accuracy of tests is important because too few isolation areas in hospitals while patients waiting for test results. Priority must be to minimise cross-infection in health-care settings" |  | Comment |  |
| 5 | "A point of care test in the ambulance to direct patients to COVID or non-COVID units; a point of care test at the front of the hospital to direct patients to COVID and non-COVID areas. (Both important; the former would be very helpful but not essential; the latter would be extremely important)." | A test for symptomatic patients presenting to a hospital to support isolation, PPE use and cohorting decisions. | Existing use case | Diagnostic |
| 6 | "Staggered testing of asymptotic hospital staff who undertake care of vulnerable patient population " | A test for potentially exposed, asymptomatic hospital workers, to support isolation, use of PPE and cohorting decisions | Existing use case | Screening |
| 7 | "Rapid test needs to be available for all the important categories- preferably within seconds!! How else do you know which bit of ED to cohort a patient into" | A test for symptomatic patients presenting to a hospital to support isolation, PPE use and cohorting decisions. | Existing use case | Diagnostic |
| 8 | "Shielded medical staff considering return to work" | A test for shielded hospital workers to inform safe return to work | New use case | Monitoring |
| 9 | "I feel all are important - another way of saying universal precautions / testing should apply " |  | Comment |  |
| 10 | "asymptomatic staff testing" | A test for potentially exposed, asymptomatic hospital workers, to support isolation, use of PPE and cohorting decisions | Existing use case | Screening |
| 11 | "definitely for hospital workers who have tested for covid 19 to return to work. I had covid 19 only symptoms persistent cough/breathlessness/no taste, no high temperature advised to return to work with cough after 7 days. other trusts policy retested before return to work and need neg result " | A test for hospital workers with a confirmed COVID-19 diagnosis to inform safe return to work | Existing use case | Monitoring |
| 12 | "testing of all staff twice a month " | A test for potentially exposed, asymptomatic hospital workers, to support isolation, use of PPE and cohorting decisions | Existing use case | Screening |
| 13 | "Testing will allow study of re-infection rates to indicate the realities of immunity and the potential value of inoculation." | A test for the general population to inform re-infection rates and immunity | New use case | Prognostic |
| 14 | "Antibody testing to see if asymptomatic patients and staff can continue to work or maintain place in society" | A test for hospital workers with a confirmed COVID-19 diagnosis to inform safe return to work | Existing use case | Monitoring |
| 15 | "antibody testing for staff or weekly swabbing if no antibodies" | A test for asymptomatic workers potentially exposed to support self-isolation decisions | Existing use case | Screening |
| 16 | "Staff antibody testing to identify those who have previously been infected" | A test for asymptomatic workers potentially exposed to support self-isolation decisions | Existing use case | Screening |
| 17 | "A test for high risk patients to see if they have previously been exposed (eg before starting chemotherapy)" | A test for patients presenting to hospital for reasons unrelated to COVID-19 to identify who can safely receive routine treatments for non-COVID-19 conditions | Existing use case | Screening |
| 18 | "Staff surveillance would be important given the reports of asymptomatic carriers" | A test for potentially exposed, asymptomatic hospital workers, to support isolation, use of PPE and cohorting decisions | Existing use case | Screening |
| 19 | "Antibody testing for staff to enable return to work and deployment to correct role" | A test for hospital workers with a confirmed COVID-19 diagnosis to inform safe return to work | Existing use case | Monitoring |
| 20 | "1. Peri-operative elective surgery is becoming a big challenge as part of RESTART programme. 2. Rapid community tests in support of high-quality public heath measures for contact tracing and isolation" | A test for patients presenting to the hospital for elective surgery | New use case | Screening |
|  |  | A test for Track and Tracing | New use case | Screening |
| 21 | "a test to determine whether a person with previous COVID would be suitable for donating immune plasma" | A test for anybody in the community with a confirmed COVID19 diagnosis to inform donation of immune plasma | New use case | Prognostic |
| 22 | "The greatest unmet need relates to the rapidity of testing rather than who needs a test - as all the groups above do. What is needed is a very rapid, accurate test, performed at the POC " |  | Comment |  |
| 23 | "Pts who may be having elective surgery which involves an AGP i.e. ENT Surgery A test for children who can then be given routine OPD audiology and ophthalmology care." | A test for patients presenting to the hospital for elective surgery | New use case | Screening |
|  |  | A test for patients presenting to hospital for reasons unrelated to COVID-19 to identify who can safely receive routine treatments for non-COVID-19 conditions | Existing use case | Screening |
| 24 | "A Rapid, Near Patient Test which would test for Influenza and SARS-CoV2 this winter would be of huge use" | A multiplex test to determine whether a symptomatic patient has been infected with COVID-19 or with influenza | New use case | Diagnosis |
| 25 | "Point of care testing for asymptomatic patients admitted as an emergency would transform the flow of patients through the hospital and especially the operating theatres. At the moment, we take full precautions with everyone which is incredibly inefficient - halves the number of operations per operating session." | A test for patients presenting to hospital for reasons unrelated to COVID-19 to identify who can safely receive emergency treatments for non-COVID-19 conditions | Existing use case | Screening |
| 26 | "At present we are able to test all who need tested so there is no unmet need for testing apart from antibody testing" |  | Not enough to extract a use case |  |
| 27 | "testing for isolated symptoms e.g. anosmia |  | Not enough to extract a use case |  |
| 28 | "Need for more sensitive tests for virus but also better understanding if positive tests at for example 30 days is dead virus" |  | Comment/ not enough to extract a use case |  |
| **CARE HOMES** | | | |  |
| **n** | **Excerpt** | **Use case** | **Comment/ existing use case** | **Final Adjudication** |
| 1 | "Testing residents prior to discharge from a short stay care setting " | A test for those about to be discharged from care homes to prevent transmission to the community | New use case | Diagnosis/ Screening |
| 2 | "Sequencing to look for strains and better understand patterns of spread in care homes" | A test to monitor the prevalence and spread of the virus in care homes | New use case | Monitoring |
| 3 | "Family members prior to visiting residents when we come out of lock down" | A test for those on admission to care homes to prevent transmission to residents | Existing use case | Screening |
| 4 | "What is needed is a test done on site with results within 30 minutes for asymptomatic staff. This would allow care homes to stay safe as the tightest risk to residents are staff." | A test for asymptomatic care home workers to support self-isolation decisions | Existing use case | Screening |
| 5 | "An antibody test would be useful for all, but only if we know that people cannot be infected more than once " | A test to determine whether asymptomatic residents have been infected previously to support isolation, PPE use, cohorting and non-COVID related treatment decisions | Existing use case | Screening |
|  |  | A test for asymptomatic care home workers to support self-isolation decisions | Existing use case | Screening |
| 6 | "Staff testing - regular testing, for example NHS couriers are tested every 4 days but care home staff only when symptomatic or the one-off whole home test. Also, Antibody testing for staff." | A test for asymptomatic care home workers to support self-isolation decisions | Existing use case | Screening |
| 7 | "Antibody test for staff " |  | Not enough to extract a use case |  |
| **General Practice** | | | |  |
| **n** | **Excerpt** | **Use case** | **Comment/ existing use case** | **Final Adjudication** |
| 1 | "A test for a patient with potential covid-19 to support anticipatory care planning conversations with relatives and likely requirements for palliative care, placement for care issues, anticipatory prescribing etc. Important for frail people. " | A test for symptomatic (frail) patients to support patient management decisions | New use case | Prognostic |
| 2 | "A test for all patients in a practice or area to find out baseline rate of infection at intervals." | A test to monitor the prevalence and spread of the virus in the community | New use case | Screening |
| 3 | "Testing of contacts of confirmed C19 cases to enable isolation if required" | A test for track and tracing | New use case | Screening |
| 4 | "good selective antibody test, to help establish potential immunity - even if the r/sh between antibodies and immunity, in terms of level and duration is not yet well known" | A test for the general population to inform re-infection rates and immunity | New use case | Prognostic |
| 5 | "The above assumes that a test is developed in future that has a very low false negative rate. Current testing is useless as a rule-out test." |  | Comment |  |
| 6 | "tests to determine if family of health care workers have had covid in order to assess their risk (and inform role allocation for staff) a test confirming prolonged immunity top priority for patients at the moment is to be able to know if someone with symptoms has covid, quickly, and for staff - have they had it in the past, and do they have covid if they get any symptoms. A test helping decide who to escalate to hospital care would be very very helpful" | A test to determine whether  relatives of care workers have previously been infected with COVID-19 to support role allocation | New use case | Screening |
|  |  | A test for the general population to inform re-infection rates and immunity | New use case | Prognostic |
|  |  | A test for symptomatic patients presenting at GP to support PPE use and isolation decisions | Existing use case | Diagnostic |
|  |  | A test potentially exposed asymptomatic GP workers to support self-isolation decisions. | Existing use case | Screening |
|  |  | A test for a patient with a confirmed COVID-19 diagnosis to help identifying who could benefit from escalation of care (e.g. hospital admission) | Existing use case | Prognostic |
| 7 | "I'm assuming these are all 'tests for COVID-19'. There are lots of other questions about 'tests' for risk stratification (FBC, CRP, d-dimer etc are pretty predictive of poor outcome at hospital admission; if that were replicated early in disease it could radically change what we do). " | A test for a patient with a confirmed COVID-19 diagnosis to help identifying who could benefit from escalation of care (e.g. hospital admission) | Existing use case | Prognostic |
| 8 | "Testing is mainly useful for public health surveillance, The problem of false negatives in pcr testing and false positives in antibody testing and the uncertainty about immunity and how long it might last , makes all the above scenarios wishful thinking as there will still be a degree of uncertainty post testing." |  | Comment |  |
| **Dental** | | | |  |
| **n** | **Excerpt** | **Use case** | **Comment/ existing use case** | **Final Adjudication** |
| 1 | "Testing should be widely available at GP clinics, Dental Practices, Pharmacies etc so patients can be screened prior to appointments at hospitals, practices and GP clinics." | A test for asymptomatic patients to support safe attendance at routine appointments | Existing use case | Screening |
| 2 | "Clinicians who had a career break should be offered both PCR and antibody tests to facilitate safe return to work." | A test for dental staff who have been potentially exposed to COVID-19 to support self-isolation decisions | Existing use case | Screening |
| 3 | "Test with immediate results that could be applied to all face to face patient contact would allow a high level of care to resumed" | A test for asymptomatic patients to support safe attendance at routine appointments | Existing use case | Screening |
| 4 | "Antibody tests for clinical staff before we return to see patients, so we can tell if we were exposed before... or after lockdown to help us decide how at risk we are." | A test for dental staff who have been potentially exposed to COVID-19 to support self-isolation decisions | Existing use case | Screening |
| 5 | "immunity testing for dental staff" | A test for dental staff who have been potentially exposed to COVID-19 to support self-isolation decisions | Existing use case | Screening |
| **Prison** | | | |  |
| **n** | **Excerpt** | **Use case** | **Comment/ existing use case** | **Final Adjudication** |
| 1 | "Clearer guidance on retesting after the initial 7 days of isolation. e.g. if it's needed. If it is needed and the patient tests positive the guidance around that. " |  | Comment |  |
| 2 | "Routine antigen testing of staff - to check if we have actually been exposed." | A test for potentially exposed, asymptomatic prison workers to support self-isolation decisions | Existing use case | Screening |
