# Supplementary material for: Unmet clinical needs for COVID-19 tests in UK health and social care settings
Source: PLoS One. 2020 Nov 12;15(11):e0242125. doi: 10.1371/journal.pone.0242125 (PMC7660574; doi:10.1371/journal.pone.0242125)
Supplement: S1 File — (DOCX) [file pone.0242125.s004.docx]

**Supporting information**

COVID-19 unmet clinical needs survey

Dear Participant,

We are working closely with national teams seeking to quickly deploy new tests for

COVID-19 across different clinical and non-clinical settings in the UK. The aim of this

survey is to better understand the unmet clinical need for medical testing in each context and to provide high quality evidence to support government policy, strategy and effective implementation.

You have been invited to participate to the survey because you are a health or social

care professional or a policy maker in UK.

You do not have to take part in the survey, and you can stop completing the survey at any point without giving a reason. A decision to withdraw at any time, or a decision not to take part, will not affect your legal rights.

The survey should take around 10-12 minutes to complete. No personal or sensitive data is collected, and your response is anonymous and will be kept confidential. No patient data are collected through the survey. There are no risks associated with taking part in this survey. Results will be disseminated through publications in scientific journals, conferences and relevant websites.

If you have any questions about the research or about the instructions at any stage,

please do not hesitate to contact: [nihr.newcastle.mic@ncl.ac.uk](mailto:nihr.newcastle.mic@ncl.ac.uk).

By clicking on the link below you consent to participate to this study.

Thank you for taking part.

Questions

1. Do you consent to participation in this survey?

- Yes
- No

Demographics and Clinical Role

1. Which region of the UK do you work in?

- Scotland
- Northern Ireland
- Wales
- North East England
- North West England
- Yorkshire and the Humber
- West Midlands
- East Midlands
- South West
- South East
- East of England
- Greater London

1. In which setting do you primarily work in?

- Care Home with nursing
- Care home without nursing
- Hospice
- Hospital-at-home
- Independent Laboratory
- General Practice
- Ambulance
- Hospital
- Primary dental care
- Secondary & community dental care
- Prison
- I don’t work in a specific clinical setting, I work in health policy [skip to Q9]

1. [IF Q3, ‘Setting…’ = ‘Hospital’] In which section(s) of the hospital do you usually work? Please tick all that are applicable

- Emergency Department
- General Medicine Ward
- Intensive Care Unit
- General Surgery Ward
- Outpatient clinic
- Laboratory
- Haematology
- Oncology
- Transplant
- Rheumatology
- Renal
- Endocrinology
- Gastroenterology and Colorectal
- Other (Please specify):

1. What is your job role? Tick all that apply

- Allied Health Professional
- Ambulance Practitioner
- Anaesthetist
- Biomedical Scientist
- Care Worker
- Clinical Scientist
- Consultant
- Dentistry
- General Practitioner
- Infectious Disease Doctor
- Intensive Care Physician
- Virologist
- Medical Doctor
- Medical Microbiologist
- Nurse
- Radiologist/Radiographer
- Respiratory Physician
- Specialist Nurse in Infection Control
- Surgeon
- Other (Please specify):

1. How many years experience do you have working in your setting?

- Less than 1 year
- Between 1 and 4 years
- Between 5 and 9 years
- 10 or more years

1. Which patient group(s) do you mostly see? Please tick all that apply.

- Neonates
- Paediatrics
- Adults
- Older people
- Not applicable

1. Have you had any involvement in the identification or care of patients with suspected or confirmed COVID-19?

- Yes
- No

1. Have you had any involvement in writing policies for the identification or care of patients with suspected or confirmed COVID-19?

- Yes
- No

Greatest unmet clinical need

Here we would like to gather your views on which roles for a new test for COVID-19 you believe to be the top priorities in relation to your setting.

Route to one of Q10 – Q17 depending on response to Q3

[IF Q3, ‘Setting…’ = ‘…health policy’], skip to Q19.

1. [IF Q3, ‘Setting…’ = ‘Ambulance’] Within an ambulance setting, which roles for a new COVID-19 test are considered the greatest unmet clinical need? Please rate the following testing roles depending on their importance to you.

|  | More Important | Important | Less Important |
| --- | --- | --- | --- |
| A test for patients requiring ambulance care for reasons unrelated to COVID19 to determine if safe for the patient to receive treatment for non-COVID conditions, and support PPE use |  |  |  |
| A test for patients requiring ambulance care for reasons related to COVID-19 to support PPE use |  |  |  |
| A test for asymptomatic paramedics to support self-isolation decisions |  |  |  |
| A test for symptomatic paramedics to support self- isolation decisions |  |  |  |
| A test for patients requiring ambulance care for reasons related to COVID-19 to identify who could benefit from hospital admission/triage to COVID-19 units |  |  |  |
| A test for paramedics with a confirmed COVID-19 diagnosis to inform safe return to work |  |  |  |
| A test to determine whether an asymptomatic patient has previously been infected with COVID-19 to support PPE use, further testing and appropriate treatment |  |  |  |
| A test to determine whether a patient with flu-like symptoms has previously been infected with COVID-19 to PPE use, support further testing and appropriate treatment |  |  |  |

Are there any relevant testing roles that we have missed? If so, please specify here (population, intended used, and treatment decision). Please also include any other comments relevant to the clinical needs for new diagnostics.

1. [IF Q3, ‘Setting…’ = ‘Care Home with nursing’ or ‘Care home without nursing’] Within a care home setting, which roles for a new COVID-19 test are considered the greatest unmet clinical need? Please rate the following testing roles depending on their importance to you.

|  | More Important | Important | Less Important |
| --- | --- | --- | --- |
| A test for those on admission to a care home to prevent transmission to existing residents |  |  |  |
| A test for residents who are asymptomatic, but have been potentially exposed, to support PPE use, isolation and cohorting decisions |  |  |  |
| A test for residents who have symptoms associated with COVID19 to support PPE use, isolation and cohorting decisions |  |  |  |
| A test for asymptomatic, care home workers who have been potentially exposed to support self-isolation decisions |  |  |  |
| A test for symptomatic care home workers to support self-isolation decisions |  |  |  |
| A test to confirm that a resident is currently infected with COVID-19 following triage testing to support treatment choices (e.g. antibiotics/antivirals) |  |  |  |
| A test for residents with a confirmed COVID-19 diagnosis to help identify who could benefit from escalation of care |  |  |  |
| A test for symptomatic residents to identify who could benefit from hospital admission |  |  |  |
| A test for residents with a confirmed COVID-19 diagnosis to inform de-escalation of care |  |  |  |
| A test to determine whether an asymptomatic resident has previously been infected with COVID-19 to support isolation, PPE use, cohorting and non-COVID treatment decisions |  |  |  |
| A test to determine whether a resident with flu-like symptoms has previously been infected with COVID-19 to support further testing, appropriate treatment, isolation and cohorting decisions |  |  |  |

Are there any relevant testing roles that we have missed? If so, please specify here (population, intended used, and treatment decision). Please also include any other comments relevant to the clinical needs for new diagnostics.

1. [IF Q3, ‘Setting…’ = ‘General Practice’] Within a General Practice setting, which roles for a new COVID-19 test are considered the greatest unmet clinical need? Please rate the following testing roles depending on their importance to you.

|  | More Important | Important | Less Important |
| --- | --- | --- | --- |
| A test for patients presenting to general practice for reasons unrelated to COVID-19 to identify those who can safely receive routine treatment for non-COVID-19 conditions |  |  |  |
| A test for symptomatic patients presenting to general practice to support PPE use and isolation decisions |  |  |  |
| A test for patients presenting to general practice who are asymptomatic but have been potentially exposed to COVID-19, to support PPE use and isolation decisions |  |  |  |
| A test for potentially exposed, asymptomatic, general practice workers to support isolation decisions |  |  |  |
| A test for symptomatic general practice workers to support isolation decisions |  |  |  |
| A test to confirm if a patient is currently infected with COVID-19 following initial testing to support treatment choices (e.g. antibiotics/antivirals) |  |  |  |
| A test for a patient with a confirmed COVID-19 diagnosis to help identify who could benefit from escalation of care (e.g. hospital admission) |  |  |  |
| A test for a patient with a confirmed COVID-19 diagnosis to inform de-escalation of care/safe discharge into care homes |  |  |  |
| A test to determine whether an asymptomatic patient has previously been infected with COVID-19 to support isolation, PPE use, and non-COVID treatment decisions |  |  |  |
| A test to determine whether a patient with flu-like symptoms has previously been infected with COVID-19 to support further testing, appropriate treatment, isolation decisions |  |  |  |

Are there any relevant testing roles that we have missed? If so, please specify here (population, intended used, and treatment decision). Please also include any other comments relevant to the clinical needs for new diagnostics.

1. [IF Q3, ‘Setting…’ = ‘Hospital’] Within a hospital setting, which roles for a new COVID-19 test are considered the greatest unmet clinical need? Please rate the following testing roles depending on their importance to you.

|  | More Important | Important | Less Important |
| --- | --- | --- | --- |
| A test for patients presenting to hospital for reasons unrelated to COVID-19 to identify who can safely receive emergency treatments for non-COVID-19 conditions |  |  |  |
| A test for patients presenting to hospital for reasons unrelated to COVID-19 to identify who can safely receive routine treatments for non-COVID-19 conditions |  |  |  |
| A test for symptomatic patients presenting to a hospital to support isolation, PPE use and cohorting decisions |  |  |  |
| A test for in-patients who are asymptomatic, but have been potentially exposed to COVID-19, to support isolation, use of PPE and cohorting decisions |  |  |  |
| A test for potentially exposed, asymptomatic hospital workers, to support isolation, use of PPE and cohorting decisions |  |  |  |
| A test for in-patients who develop new clinical features of COVID-19 (e.g. on chest imaging) during their hospital stay to support isolation, use of PPE and cohorting decisions |  |  |  |
| A test for symptomatic hospital workers to support self-isolation decisions |  |  |  |
| A test to confirm that a patient is currently infected with COVID-19 following initial testing to support treatment choices (e.g. antibiotics/antivirals) |  |  |  |
| A test for in-patients with a confirmed COVID-19 diagnosis to help identify who could benefit from escalation of care (e.g. vulnerable, at high risk, severe) |  |  |  |
| A test for in-patients with a confirmed COVID-19 diagnosis to inform de-escalation of care/safe discharge into care homes |  |  |  |
| A test for hospital workers with a confirmed COVID-19 diagnosis to inform safe return to work |  |  |  |
| A test to determine whether an asymptomatic patient has previously been infected with COVID-19 to support isolation, PPE use, cohorting and non-COVID treatment decisions |  |  |  |
| A test to determine whether a patient with flu-like symptoms has previously been infected with COVID-19 to support further testing, appropriate treatment, isolation and cohorting decisions |  |  |  |

Are there any relevant testing roles that we have missed? If so, please specify here (population, intended used, and treatment decision). Please also include any other comments relevant to the clinical needs for new diagnostics.

1. [IF Q3, ‘Setting…’ = ‘Hospital-at-home’] Within a hospital-at-home setting, which roles for a new COVID-19 test are considered the greatest unmet clinical need? Please rate the following testing roles depending on their importance to you.

|  | More Important | Important | Less Important |
| --- | --- | --- | --- |
| A test for patients with medical issues unrelated to COVID-19 to identify who can safely receive emergency treatments for non-COVID-19 conditions |  |  |  |
| A test for patients with medical issues unrelated to COVID-19 to identify who can safely receive routine treatments for non-COVID-19 conditions |  |  |  |
| A test for patients who are asymptomatic to support isolation and use of PPE |  |  |  |
| A test for symptomatic patients to support isolation and use of PPE |  |  |  |
| A test for potentially exposed, asymptomatic, hospital-at-home workers to support self-isolation decisions |  |  |  |
| A test for symptomatic hospital-at-home workers to support self-isolation decisions whilst waiting for confirmatory testing |  |  |  |
| A test to confirm that a patient is currently infected with COVID-19 following initial testing to support treatment choices (e.g. antibiotics/antivirals) |  |  |  |
| A test for patients with a confirmed COVID-19 diagnosis to help identify who could benefit from escalation of care (i.e. hospital admission) |  |  |  |
| A test for patients with a confirmed COVID-19 diagnosis to inform de-escalation of care/discharge from hospital decisions |  |  |  |
| A test for patients with a confirmed COVID-19 diagnosis to inform de-isolation decisions |  |  |  |
| A test for hospital-at-home workers with a confirmed COVID-19 diagnosis to inform safe return to work |  |  |  |
| A test to determine whether an asymptomatic patient has previously been infected with COVID-19 to support isolation, PPE use, cohorting and non-COVID treatment decisions |  |  |  |
| A test to determine whether a patient with flu-like symptoms has previously been infected with COVID-19 to support further testing, appropriate treatment, isolation and cohorting decisions |  |  |  |

Are there any relevant testing roles that we have missed? If so, please specify here (population, intended used, and treatment decision). Please also include any other comments relevant to the clinical needs for new diagnostics.

1. [IF Q3, ‘Setting…’ = ‘Hospice’] Within a hospice setting, which roles for a new COVID-19 test are considered the greatest unmet clinical need? Please rate the following testing roles depending on their importance to you.

|  | More Important | Important | Less Important |
| --- | --- | --- | --- |
| A test for symptomatic patients to support isolation and PPE use |  |  |  |
| A test for potentially exposed asymptomatic patients, to support isolation and PPE use |  |  |  |
| A test for potentially exposed, asymptomatic hospice workers, to support isolation, use of PPE |  |  |  |
| A test for symptomatic hospice workers to support self-isolation decisions |  |  |  |

Are there any relevant testing roles that we have missed? If so, please specify here (population, intended used, and treatment decision). Please also include any other comments relevant to the clinical needs for new diagnostics.

1. [IF Q3, ‘Setting…’ = ‘Primary dental care’ or ‘Secondary & community dental care’] Within a dental care setting, which roles for a new COVID-19 test are considered the greatest unmet clinical need? Please rate the following testing roles depending on their importance to you.

|  | More Important | Important | Less Important |
| --- | --- | --- | --- |
| A test for asymptomatic patients to support safe attendance at routine appointments |  |  |  |
| A test for symptomatic patients to support safe attendance at routine dental appointments |  |  |  |
| A test for asymptomatic patients to support safe attendance at urgent dental appointments to guide the appropriate use of PPE and aerosol generating procedures |  |  |  |
| A test for symptomatic patients to support safe attendance at urgent dental appointments to guide the appropriate use of PPE and aerosol generating procedures |  |  |  |
| A test for dental staff who are asymptomatic, but have been potentially exposed to COVID-19, to support use of PPE and self- isolation |  |  |  |
| A test for symptomatic dental staff to support self-isolation decisions |  |  |  |
| A test for dental staff with a confirmed COVID-19 diagnosis to inform safe return to work |  |  |  |
| A test to determine whether an asymptomatic patient has previously been infected with COVID-19 to support isolation and PPE use |  |  |  |
| A test to determine whether a patient with flu-like symptoms has previously been infected with COVID-19 to support escalation of care advice |  |  |  |

Are there any relevant testing roles that we have missed? If so, please specify here (population, intended used, and treatment decision). Please also include any other comments relevant to the clinical needs for new diagnostics.

1. [IF Q3, ‘Setting…’ = ‘Prison’] Within a prison setting, which roles for a new COVID-19 test are considered the greatest unmet clinical need? Please rate the following testing roles depending on their importance to you.

|  | More Important | Important | Less Important |
| --- | --- | --- | --- |
| A test for those on admission to a prison to prevent transmission to existing residents |  |  |  |
| A test for prisoners who are asymptomatic, but have been potentially exposed, to support isolation and cohorting |  |  |  |
| A test for prisoners who have symptoms associated with COVID-19 to support PPE use, isolation and cohorting decisions |  |  |  |
| A test for potentially exposed, asymptomatic, prison workers to support self-isolation decisions |  |  |  |
| A test for symptomatic prison workers to support self-isolation decisions |  |  |  |
| A test to confirm that a prisoner is currently infected with COVID-19 following initial testing to support treatment choices (e.g. antibiotics/antivirals) |  |  |  |
| A test for prisoners with a confirmed COVID-19 diagnosis to help identify who could benefit from escalation of care |  |  |  |
| A test for prisoners with a confirmed COVID-19 diagnosis to inform de-escalation of care |  |  |  |
| A test to determine whether an asymptomatic prisoners has previously been infected with COVID-19 to support isolation, PPE use, cohorting and non-COVID treatment decisions |  |  |  |
| A test to determine whether a prisoner with flu-like symptoms has previously been infected with COVID-19 to support further testing, appropriate treatment, isolation and cohorting decisions |  |  |  |

Are there any relevant testing roles that we have missed? If so, please specify here (population, intended used, and treatment decision). Please also include any other comments relevant to the clinical needs for new diagnostics.

Other clinical settings

We are now interested about your views on settings different from your own.

Here is a list of settings that could benefit from a COVID-19 diagnostic test:

Hospital

Hospice

Hospital-at-home

Ambulance

Care home

Prison

GP medical practice

Primary (general) dental care

Secondary dental care

Domestic Residence (i.e. patient/key worker's home)

1. Do you think any of those of the list should be prioritised over your setting?

- Yes [skip to Q19]
- No [skip to Q20]

1. Which of the following settings should be prioritised?

- Hospital
- Hospital-at-home
- Ambulance
- Hospice
- GP medical practice
- Care home
- Prison
- Primary (general) dental care
- Secondary dental care
- Domestic residence (e.g. individual's home)

COVID-19 diagnostic tests

1. Are COVID-19 diagnostic test(s) currently used in your clinical setting?
   - Yes [skip to Q22]
   - No [skip to Q21]
2. What are the consequences of there being no COVID-19 tests available in your setting? Tick all that apply.

- Longer length of (hospital/ICU) stay
- Inappropriate hospital referrals
- Inappropriate hospital admission
- Inappropriate hospital discharge
- Inappropriate treatment
- Patient anxiety/discomfort
- Inappropriate use of isolation
- Inappropriate cohorting
- Misuse of antibiotics and potential increase of antibiotic resistance in the population
- Inappropriate use of PPE
- Delays in administering treatments to patient
- Delays in escalating treatments to patient
- Differences in case reporting across health care facilities
- Potential spread of infection
- Other (please specify)

COVID-19 Diagnostics: Current Practice

1. Please select the type(s) of COVID-19 diagnostic test(s) that is/are currently used in your clinical setting.

Laboratory Point of Care

Serology (e.g. blood test) □ □

Molecular (e.g. swab test) □ □

Other, please specify:

1. For each test above, can you please indicate which safety precautions were taken. Tick all those apply

| Test | None | Standard COVID-19 PPE | BSL2 | BSL3 | Not sure |
| --- | --- | --- | --- | --- | --- |
| Serology - laboratory |  |  |  |  |  |
| Serology- POCT |  |  |  |  |  |
| PCR based - laboratory |  |  |  |  |  |
| PCR based - POCT |  |  |  |  |  |

Problems with Diagnosing COVID-19 (part 1)

1. In your view, are there any problems with current COVID-19 testing practices in your clinical setting?

- Yes
- No [skip to Q27]
- Don’t know

Problems with Diagnosing COVID-19 (part 2)

1. In your view, what are the current problems with the diagnostic tests that are available to you in your setting? Please rank the following options in terms of importance, or select not applicable

|  | **More important** | **Important** | **Less important** | **Not applicable** |
| --- | --- | --- | --- | --- |
| Necessary equipment is not readily available |  |  |  |  |
| Availability of  reagents |  |  |  |  |
| Long turnaround time |  |  |  |  |
| Insufficient throughput |  |  |  |  |
| Difficulty in obtaining a sample |  |  |  |  |
| Patient acceptability of sampling technique |  |  |  |  |
| Patient safety during testing procedure |  |  |  |  |
| Staff safety during testing procedure |  |  |  |  |
| Technically complex/time-consuming to perform the test |  |  |  |  |
| Too expensive |  |  |  |  |
| Test usability (too large/too heavy/not user friendly) |  |  |  |  |
| Poor ability to confirm if a patient has COVID-19 |  |  |  |  |
| Poor ability to confirm if a patient does not have COVID-19 |  |  |  |  |
| Difficult to interpret test results |  |  |  |  |
| Excessive test failure rate |  |  |  |  |
| Lack of standardised technique (e.g. different method of sampling, period of incubation) |  |  |  |  |
| Lack of established protocols to inform decision making after positive/negative results |  |  |  |  |

1. In relation to the diagnostic tests that are available to you in your clinical setting, what are the consequences of these problems? Tick all that apply.

- Longer length of (hospital/ICU) stay
- Inappropriate hospital referrals
- Inappropriate hospital admission
- Inappropriate hospital discharge
- Inappropriate treatment
- Patient anxiety/discomfort
- Inappropriate use of isolation
- Inappropriate cohorting
- Misuse of antibiotics and potential increase of antibiotic resistance in the population
- Inappropriate use of PPE
- Repeat testing required
- Delays in administering treatments to patient
- Delays in escalating treatments to patient
- Differences in case reporting across health care facilities
- Potential spread of infection
- Other (please specify)

1. How did you hear about this survey? Please tick all that apply

- Social Media
- Word of mouth
- Email
- Webpage
- Other (please specify)

1. Is there any additional feedback that you would like to provide?
2. Do you consent to submit this survey?

- Yes
- No
