# Supplementary material for: Unmet clinical needs for COVID-19 tests in UK health and social care settings
Source: PLoS One. 2020 Nov 12;15(11):e0242125. doi: 10.1371/journal.pone.0242125 (PMC7660574; doi:10.1371/journal.pone.0242125)
Supplement: Attachment — Submitted filename: PLOS ONE- Unmet clinical needs for COVID.docx [file pone.0242125.s005.docx]

Unmet clinical needs for COVID-19 tests in UK health and social care settings

Sara Graziadio, Samuel G. Urwin, Paola Cocco, Massimo Micocci, Amanda Winter1, Yaling Yang4

, D. Ashley Price, Mike Messenger, A. Joy Allen &, Bethany Shinkins.

1 NIHR Newcastle In Vitro Diagnostics Co-operative, Newcastle upon Tyne Hospitals Foundation Trust

2 NIHR Leeds In Vitro Diagnostics Co-operative, Leeds Teaching Hospitals NHS Trust

3NIHR London In Vitro Diagnostics Co-operative, Imperial College London

4NIHR Community Healthcare MedTech and In Vitro Diagnostics Co-operative Nuffield Department of

Primary Care Health Sciences, University of Oxford

5 Leeds Centre for Personalised Health and Medicine, University of Leeds

6 NIHR Newcastle In Vitro Diagnostics Co-operative, Newcastle University

7 Test Evaluation Group, Institute for Health Sciences, University of Leeds

¶ These authors contributed equally

& ¶ These authors also contributed equally

* Corresponding author

A. Joy Allen

Correspondence: [Joy.Allen@newcastle.ac.uk](mailto:Joy.Allen@newcastle.ac.uk)

ABSTRACT

There is an urgent requirement to identify which clinical settings are in most need of COVID-19 tests, and the priority role(s) for tests in these settings to accelerate development of tests fit for purpose in health and social care across the UK. This study sought to identify and prioritise unmet clinical needs for COVID-19 tests across different settings within the UK health and social care sector via an online survey of health and social care professionals and policy makers. Four hundred and forty-seven responses were received between 22nd May and 15th June 2020. Hospitals and care homes were recognised as the settings with the greatest unmet clinical need for COVID-19 diagnostics, despite reporting more access to laboratory molecular testing than other settings. Hospital staff identified a need for diagnostic tests for symptomatic workers and patients, whereas care home staff expressed an urgency for screening at the front door to protect high risk residents and limit transmission. Length of time to test result was considered a widespread problem with current testing across all settings. Rapid tests for staff were regarded as an area of need across general practice and dental settings, alongside tests to limit the use of antibiotics.

INTRODUCTION

Testing is playing an integral role in the international response to the current coronavirus pandemic,

facilitating national surveillance and guiding patient management. Testing strategies have been

heavily scrutinised, and it is widely acknowledged that the tests currently available are far from ideal.

For example, reverse transcription polymerase chain reaction (RT-PCR) based tests, the most

commonly used laboratory-based tests for determining current infection, has a false negative rate of

around 20% (depending on symptom duration, sampling technique and site),[1].

The industry response has been unprecedented; by the 17th July 2020, 746 tests had been developed

or are under development,[2]. Although this level of innovation offers hope that the current testing

shortfalls can be overcome; this creates a real challenge for diagnostic regulatory bodies. For many

years, the conversion of diagnostic innovation from bench to clinical practice has been riddled with

problems and is notoriously slow,[3]. A rapid evaluation pipeline is required to ensure that tests

offering real health benefits are integrated into practice within a timeframe that will support

international efforts to curtail the transmission of SARS-CoV-2.

In March 2020, the World Health Organization (WHO) outlined a research roadmap recommending

the development of Target Product Profiles (TPPs) to drive the research and innovation process

around new diagnostic tests for COVID-19,[4]. A TPP is a document that summarises in advance the

desirable and minimally acceptable specifications for a new test to address a well-defined clinical

need. The overarching aim is to ensuring that innovation efforts are focused on developing ‘fit for

purpose’ tests,[5-6]. The National Institute for Health and Care Excellence (NICE) have recently begun

an economic modelling exercise to help inform TPP specifications for COVID-19 tests,[7].

At the core of TPP development is the scoping and definition of unmet clinical need,[5]. Tests often

have numerous roles in clinical practice across multiple clinical settings (e.g. primary and secondary

care) and positions in a care pathway (e.g. to diagnose or to monitor disease),[8]. There is an urgent

need to identify which health and social care settings are most in need of COVID-19 tests, and the

priority role(s) for tests in these settings, particularly ahead of the winter season where other

respiratory viral like illnesses will also be in circulation.

To this end, the National Institute for Health Research (NIHR) MedTech and In Vitro Diagnostics (IVD)

Co-operatives (MICs), on behalf of the COVID-19 National DiagnOstic Research and Evaluation

Platform (CONDOR),[9], designed an online survey at pace to gather opinion from health and social

care professionals and policymakers on unmet clinical needs for COVID-19 tests in the UK. The primary

objective of the survey was to determine which settings are in most need of a new COVID-19 test and

determine the priority role(s) for new tests within each setting. The results will be used to help prioritise and refine TPP development, plan for winter allocation of tests and to inform economic modelling carried out by the NICE diagnostics assessment programme. Interim reports were shared with the Department of Health and Social Care (DHSC) and NICE on 29/05/2020 and 28/06/2020 to inform decisions around UK national testing policies.

MATERIALS AND METHODS

The development and reporting of the survey followed good practice guidelines published by Kelley et al.,[10] and highlighted by the Enhancing the QUAlity and Transparency Of health Research (EQUATOR) network,[11]. The survey questions were written based on an initial list of priority use cases developed by the DHSC COVID-19 Test Approvals Groups.

**Survey design, development and dissemination**

Targeted respondents of this survey were health and social care professionals working in the UK across a variety of settings, and policymakers. The survey was voluntary and anonymised, with no personal, sensitive or patient data collected. All respondents were asked to select which setting was most in need of a novel COVID-19 test, and then health and social care professionals were asked to rate the importance of different use cases specific to the setting where they worked. The use cases described potential uses and roles of tests at specific points in the pathway; they were technology agnostic, but described roles that could be fulfilled by molecular or serology tests. All respondents, apart from policymakers, were asked if diagnostic testing for COVID-19 was available in the setting in which they worked and, if so, they were asked which type of tests (e.g. molecular, serological) were available and whether they perceived any problems with the tests.

A first draft of the questionnaire was developed using Online surveys (formerly BOS),[12], with the support of two clinicians, and then the questionnaire was tested and revised by the wider authorship team to improve clarity and flow. The survey was then piloted by six hospital clinicians (one of whom worked closely with the ambulance service and another with care homes) and one dentist before its launch, with feedback incorporated into the final version of the survey. Piloting the survey identified problems with wording, and confirmed whether the survey functioned as intended in terms of access, navigation and submission, [13-14]. Pilot data were not included in the final analysis to avoid contamination of results, [14]. 5 The survey was launched and disseminated via a variety of networks including the NIHR Clinical Research Network (CRN) Coordinating Centre networks, clinical contacts of the NIHR MICs, as well as social media (limited to Twitter and LinkedIn, to our knowledge). A copy of the survey is available in the Supplementary Material.

**Survey analysis**

A setting was required to have at least 30 respondents in order to be included in the analysis, except for policymakers, where a smaller sample size was anticipated due to policymakers comprising a smaller proportion of the workforce compared to health and social care professionals. Data processing and analysis were primarily conducted in the statistical programming language R,[15], with qualitative free text responses analysed in Microsoft Excel. Where appropriate, combined heat map and dendrograms were used to visualise results and identify similarities/differences between settings using the R function ‘heatmap.2’ from the ‘gplots’ package,[16]. Hierarchical clustering was performed using the complete agglomeration method, and the Euclidean method was used to calculate the distances between the data. Respondents selected whether they felt each use case in their specific setting was ‘More Important’, ‘Important’ or ‘Less Important’ relative to the others in the list. A scoring system was applied (More Important = 1, Important = 0, Less Important = -1), with the mean score and standard deviation calculated to summarise responses. The use cases were then ranked from the highest mean score to the lowest. A framework for categorising use cases was developed to facilitate interpretation of results. Use cases were grouped in the survey based on their intended use: ‘Screening’ (to support isolation, personal protective equipment (PPE) use and cohorting decisions), ‘Diagnostic’ (to inform both infection control and treatment stratification), ‘Prognostic’ (escalation of care decisions) and ‘Monitoring’ (deescalation of care and safe return to work),[17]. These were subsequently validated and independently re-grouped. In case of disagreement, a single arbiter resolved discrepancies. In the survey, the respondents could also suggest additional use cases via a free text field, if they perceived them to be missing from the provided list. Based on the previously defined framework, additional use cases were analysed to identify responses that were similar to the provided list, then independently assigned to the ‘Screening’, ‘Diagnostic’, ‘Prognostic’ and ‘Monitoring’ categories.

**Patient and public involvement**

Seven members of the CONDOR,[9] patient and public representative panel were invited to participate in a focus group discussion. The background, methods and results of the survey were presented to the 6 group, who were then invited to comment on these aspects. The participants provided verbal and written feedback during and after the meeting which has been incorporated into the discussion and will form the basis of a lay summary for the public to be published on the CONDOR website,[9]. They agreed that the survey addressed important questions in relation to COVID-19 diagnostics. They raised the issues of geographical and ethnic representativeness of sampling, the potential omission of settings and the importance of including patient experiences in future surveys. This included the impact of testing on their physical and mental health and the overall confidence of the public in COVID19 testing.

RESULTS

The survey was launched at 4pm on the 22nd May 2020 and was open until 9am on the 15th June 2020, receiving 447 completed responses (Table 1). It was not possible to estimate the number of potential respondents reached due to the variety of dissemination routes used. The distribution of survey responses over time, grouped by the setting in which the respondents worked, is presented in Supplementary fig. 1.

*Table 1 Characteristics of survey respondents*

**Demographics of respondents.**

The majority of the respondents were from the North East of England (n=217, 48.6%), with 411 respondents in total from England (92%) (Table 1). The most common setting in which respondents worked was hospitals (n=189, 42%), followed by primary dental care (n=65, 15%) and general practice (n=55, 12%) (Table 1). The hospice (n=14, 3%), ambulance service (n=9, 2%) and hospital-at-home settings (n=3, 1%) received less than 30 respondents.

**Unmet clinical need**

Settings which were deemed the highest priority were hospitals (Figure 1, 266 selections across all settings, 55.8% of respondents) and care homes (196 selections across all settings, 41.1% of respondents). This finding was apparent even when considering those who didn’t select their own setting (the red bars in Figure 1). All other settings received less than 100 selections each. Twelve policymakers completed the survey. Most of them prioritised care homes (n=4), followed by domestic residences (n=3) and GP practices (n=2), with hospitals, hospices and the ambulance service selected by one respondent each. Care homes were generally prioritised by respondents working in other settings.

*Figure 1. Settings with the greatest perceived unmet clinical need for COVID-19 tests, showing votes from respondents for their own setting (blue shading) and other settings (red shading).*

Use case ranking was explored in care homes with and without nursing independently, and the results were similar, as were those for primary and secondary dental care. We then pooled results from each of these two settings in order to increase the sample size for this and subsequent analyses. The top three clinical needs for hospitals (Table 2) were all related to diagnosis to support infection control decisions for patients presenting to hospital (1st), for workers (2nd), and for in-patients (3rd). For care homes, screening tests to support infection control decisions were the most important, with a test for those at admission and workers ranked first and second, respectively, and a test for patients potentially exposed ranked third. Respondents from GP and dental settings showed a similar focus on infection control measures for workers, identifying the two most important needs as testing to support screening and diagnosis of workers. The third most important identified need was around monitoring for both settings, where the dental setting showed a preference for a test to support safe return to work and the GP setting a test to safely discharge patients into care homes. Respondents from the prison setting preferred a test to identify prisoners with COVID-19 at admission with symptoms (1st), and those with infection who are asymptomatic (2nd). The third most important identified need was to support workers in their self-isolation decisions when symptomatic, however this had a much lower score than the first two ranked needs. No respondents across settings seemed to consider the few prognostic use cases proposed as being particularly important.

***Table 2 Unmet clinical need priorities in each setting from respondents who worked in those settings, ranked by mean score from highest (most important clinical need) to lowest (least important clinical need). Table key: ‘PPE’ = ‘personal protective equipment’, x indicates use cases that were not proposed 9 for that setting. The three most important use cases identified by the respondents in each setting are indicated in red.***

Some respondents suggested additional needs for clinical tests for COVID-19. While 32 (66.6%) of these could be mapped to existing use cases (the majority of which were for screening tests, n=6 for patients, n=14 for staff and n=4 for community screening), 16 (33.3%) were judged as new use cases by the authors (Supplementary Tables 1 and 2). Those that could not be mapped to existing use cases, highlighted the following additional needs: tests for ‘track and trace’, to detect immunity, to distinguish infective cases from non-active infections, to allow safe delivery of elective surgeries, and multiplex tests (i.e. panel tests that can detect multiple respiratory pathogens at the same time) for the winter season. Antibody tests (i.e. serology testing) were mentioned 12 times, with the majority of respondents suggesting that antibody tests would be useful to understand who had COVID-19, whilst some also recognised the uncertainty around antibody testing.

**Problems with medical tests and their availability**

The settings where the respondents most frequently stated that COVID-19 tests were available (Figure 2) were hospitals (87%), prisons (85%) and care homes (78%). The settings where the respondents most frequently stated that no COVID-19 tests were available were dental care (86%) and GP (74%). In all settings where COVID-19 testing was reported as being available, molecular laboratory testing was the most common option. Serology point of care tests were only available in GPs and hospitals.

***Figure 2.*** *The availability of COVID-19 tests in each setting as reported by respondents. Primary and secondary dental settings were merged, in addition to care homes with and without nursing. The ‘Hospital-at-home’ (n=3), hospices (n=14) and ambulance setting (n=9) were excluded due to the small number of overall respondents. Respondents were able to select multiple answers to this question. Plot key: ‘GP’ = ‘General Practice’; ‘POC’ = ‘Point of care’; ‘lab’ = ‘Laboratory.*

Respondents who stated that there were no COVID-19 tests available most frequently reported (Figure 3) that ‘Potential spread of infection’ was the consequence of the lack of COVID-19 testing in the dental setting ‘Delays in administrating treatments to patient’ was the most frequently reported response. The hierarchical cluster analysis suggested that the perceived consequences of the lack of COVID-19 testing within the dental and GP settings were similar, where the misuse of antibiotics/antibiotic resistance reported frequently Within the prison setting, there were lower numbers reporting hospital and treatment-associated consequences.

***Figure 3****. Perceived consequences of no COVID-19 tests being available in each setting. Primary and secondary dental settings were merged, in addition to care homes with and without nursing. The ‘Hospital-at-home’ (n=3), hospice (n=14) and ambulance setting (n=9) were excluded due to the small 10 number of overall respondents. A hierarchical cluster analysis indicates how similar/dissimilar the perceived consequences of no COVID-19 tests are between settings. The numbers of responses from individual settings for this particular question are displayed on the x axis (n=x). The histogram in the top left of the plot area shows the distribution of values, and provides a key to indicate the specific colour shade of different values.*

Across the majority of settings, most respondents stated that there were problems with testing in the setting where they worked (Figure 4). ‘Long turnaround time’ for results of COVID-19 tests was reported most frequently as the most important problem across all settings, whereas ‘Test usability’ and ‘Too expensive’ were the least reported problem (Figure 5). There were similarities in the most important reported problems between hospital and GP respondents, with the second most frequent problem in the hospital and GP setting being the ‘Poor ability to confirm if a patient does not have COVID-19’ (sensitivity), closely followed by the ‘Poor ability to confirm if a patient has COVID-19 (specificity). Prison respondents reported a ‘Lack of established protocols to inform decision making after positive/negative results’ as their most important problem. Responses from care homes and dental settings were also similar, with issues around availability of equipment. ‘Difficulty in obtaining a sample’ was reported more frequently for care homes than other settings.

**Figure 4.** Perception of problems associated with COVID-19 testing practices in each setting. Primary and secondary dental settings were merged, in addition to care homes with and without nursing. The ‘Hospital-at-home’, hospice and ambulance setting were excluded due to the small number of overall respondents (n<15). Respondents selected a single 'Yes', 'No' or 'Don't know' answer. The number of

responses from individual settings for this particular question is displayed on the y axis (n=x).

**Figure 5.** Most important perceived problems associated with COVID-19 testing in each setting. Primary and secondary dental settings were merged, in addition to care homes with and without nursing. The ‘Hospital-at-home’, hospice and ambulance setting (n<15) were excluded due to the small

number of overall respondents. A hierarchical cluster analysis indicates how similar/dissimilar the

perceived problems associated with COVID-19 testing are between settings. The numbers of responses

from individual settings for this particular question are displayed on the x axis (n=x). The histogram in

the top left of the plot area shows the distribution of values, and provides a key to indicate the specific

colour shade of different values.

The most frequent consequence associated with these problems (Figure 6) was consistently reported as the ‘Potential spread of infection’ along with ‘Patient anxiety/discomfort’ especially within the GP setting and care homes, and ‘Inappropriate use of PPE’ in the dental setting. ‘Repeat testing 11 requirement’ in prisons and GP settings was also reported as a frequent consequence of these problems.

**Figure 6.** Perceived consequences related to the problems associated with COVID-19 testing in each

setting. Primary and secondary dental settings were merged, in addition to care homes with and

without nursing. The ‘Hospital-at-home’, hospice and ambulance setting (n<15) were excluded due to

the small number of overall respondents. A hierarchical cluster analysis indicates how

similar/dissimilar the perceived consequences related to the problems associated with COVID-19

testing are between settings. The numbers of responses from individual settings for this particular

question are displayed on the x axis (n=x). The histogram in the top left of the plot area shows the

distribution of values, and provides a key to indicate the specific colour shade of different values.

DISCUSSION

To our knowledge, this is the first survey of health and social care professionals and policymakers to identify and prioritise unmet clinical needs for medical tests for COVID-19. We focused the survey within the UK health and social care system and covered a range of settings. Results of this survey informed the recent guidelines in UK around testing for COVID-19 in various settings and is informing NICE early economic modelling. These results are important for planning policies for the next winter season when multiple respiratory conditions will be present and diagnostic tests will be crucial for the correct identification of COVID-19 patients.

Unmet clinical need for testing

Hospital and care home settings (with and without nursing) were prioritised as being most in need of novel COVID-19 tests. Hospitals and care homes with nursing were reported as having greater access to testing compared with other settings, although care homes without nursing had very limited access to testing, highlighting a clear unmet clinical need. The data suggested that hospitals were in most need of diagnostic tests (i.e. for symptomatic individuals), whereas respondents from care homes require tests to screen asymptomatic individuals. These differing roles were important for both patients and care workers across these settings. The current testing policy in UK hospitals, implemented on the 5 th June 2020, of testing every individual with potential COVID-19 symptoms,[18], addresses this clinical need (the last hospital response to the survey was on the 3 rd June 2020). The more extensive need for tests in care homes appears to reflect the increased risk for older people,[19], where early identification of COVID-19 is extremely important for appropriate use of isolation (to decrease the risk of infection transmission) and treatment (to decrease the risk of hospital admission or death), the two major concerns associated with the lack of availability of testing. Although it is important for care home residents to safely receive visitors, the overall risk to wider care home residents needs to be minimal, therefore highlighting the importance of early identification of new cases and appropriate isolation use. Very recently (27th July 2020,[20]), the policy for UK care homes changed, requiring weekly testing of staff and monthly testing of residents. This is in line with the clinical need identified through this survey but might pose a challenge in terms of test provision. In this context, pooling of resident samples for testing might be a reasonable solution for screening in low prevalence conditions,[21]. When a positive pool is detected, individual testing of residents and staff might follow.

Care homes reported issues with patient discomfort and difficulty in obtaining samples (more than others). There is therefore a need to develop tests which use sample types which are more easily obtained. Multiplex tests could also be useful in this population in the winter season, where the detection of multiple viruses is made possible through collection of a single sample.

The respondents from the prison setting indicated there was good availability of tests, with similar needs to the hospital setting for diagnosing and screening people at the front door to limit transmission of the virus to other prisoners. They did not have access to serology tests but they seemed satisfied with the tests they had available, except for the long time to results and the lack of clear protocols to guide interpretation and actions following the results. Guidelines and care pathways should be developed in collaboration with health and social care professionals working in prisons to provide effective support during the winter season. This could help address health inequalities which have been highlighted by the COVID-19 pandemic,[22].

Interestingly, general practice and dental settings had similar needs which were mainly focused on identifying workers with COVID-19 (symptomatic or asymptomatic) who could transmit the disease to individuals in the community. Comparatively, these settings had limited access to COVID-19 tests and the main highlighted consequence of this was the inappropriate use of antibiotics. The most common new use cases stipulated by the respondents included tests that could support the Track and Trace program, surveillance of the general population as well as re-infection rates. Since policy decisions to limit or enhance lockdown measures are mainly based on these numbers,[23], there is a need to increase testing capacity in these settings.

Across all settings two main themes emerged. The first was the concern for the potential consequences of infection transmission, followed by the concerns around the long turnaround time for test results,[24] to inform isolation decisions and PPE use. The role of treatment stratification fo diagnostic tests might become more important in the future, once the clinical evaluation of treatments for COVID-19 are concluded (e.g. REMAP-CAP, [25])’

**Limitations**

There were limitations in the design of the survey. The survey was developed at pace, and as a result, could have benefitted from more comprehensive piloting, encompassing respondents from all targeted settings. The results had to be swiftly reported to inform government testing decisions, and therefore the survey was only open for a limited period, which resulted in small sample sizes for some settings. Results for hospitals, care homes, general practices and dental settings are likely to be more robust compared to other settings. Rapid development and dissemination of the survey was required to capture the current picture of a fast-moving landscape, and may not have been optimal methodologically, however, it ensured increased impact by informing decision makers. Furthermore, the number of misinterpreted use cases was low ranging from 5 and 11% of the total number of respondents in each setting, and also the percentage of new use cases that were not included was low (0-11%). This seems to indicate good identification and clear wording of the use cases; the higher percentage of missed and unclear use cases reported by GP respondents (11%) may reflect the lack of piloting in that setting. The survey was intended for response by health and social care professionals, although no questions were incorporated into the survey to explicitly validate the inclusion criteria. Questions to identify high risk staff subgroups were not included. Settings were targeted for dissemination at different times whilst the survey was open (see Supplementary fig. 1 for the distribution of responses over time). The responses received may reflect changing clinical needs due to the fluctuating prevalence of COVID-19 and the different availability of tests during the pandemic. However, the survey was only open a short time covering three weeks at the tail end of the first wave of the pandemic in the UK, so these effects are likely to be small. The majority of the respondents worked in hospitals and in the North East of England, so results are less representative of other settings and other regions of the UK. Finally, a limitation of qualitative research is that people who have a strong opinion on the subject are often the ones most likely to participate, potentially introducing selection bias. These limitations should be taken into account when inferring any conclusions from the data presented.

**Conclusions and next steps**

In this first assessment of clinical need for COVID-19 tests among experts in the UK, long turnaround of results was the most commonly reported problem across all settings in May/June 2020, the consequence of which was identified as potential transmission of infection. This would suggest a need for more rapid testing across all settings. Since it is unlikely that rapid near patient testing will be available for the coming winter season to screen everyone, more work is needed to refine the higher priority groups, and to understand the role of multiplex tests in the coming winter season. From the current work, the groups with highest need for COVID-19 testing were identified as (in order): staff and patients with symptoms of COVID-19 in hospitals; screening of staff, visitors and new residents in care homes; staff in general practice and dental settings; screening of new prisoners, visitors, existing prisoners and workers with symptoms of COVID-19. These needs are most likely to be met by molecular tests, which are most accurate soon after the patient becomes infective,[26]. Antibody tests seem less useful until the biology of the disease and its relationship with immunoglobulin A (IgA), G (IgG) and M (IgM) antibodies is clearer,[27]. In care homes, quick and cheap tests with lower accuracy but frequent testing might be appropriate for screening, as suggested by modelling,[28]. Alternatively, when prevalence is low, pooling of samples from residents could be a sensible approach for screening residents,[21]. Rapid tests (under 10 minutes) that help to distinguish viral from bacterial infections might be also useful in GP and dental setting to limit antibiotic resistance in the community. Despite more work needed to fully characterise the unmet clinical need, it seems likely that multiplex tests will be useful in the next winter season when other respiratory viruses will be circulating. Particularly in care homes and in older populations of patients in order to optimize sample collection.
