# Supplementary material for: Unmet clinical needs for COVID-19 tests in UK health and social care settings
Source: PLoS One. 2020 Nov 12;15(11):e0242125. doi: 10.1371/journal.pone.0242125 (PMC7660574; doi:10.1371/journal.pone.0242125)
Supplement: Attachment — Submitted filename: Response to Reviewers.docx [file pone.0242125.s006.docx]

**Newcastle In Vitro Diagnostics Co-operative**

M2.088 2^nd^ Floor

William Leech Building,

Medical School,

Newcastle University,

Framlington Place,

Newcastle upon Tyne

NE2 4HH

21^th^ October 2020

Dear Editors,

Thank you and the reviewers for your extremely positive and helpful reviews of our paper: ‘Unmet clinical needs for COVID-19 tests: A survey of UK health and social care professionals’.

- We have addressed the grammatical concerns of reviewer 1 and made significant amends to the manuscript according to comments on the returned manuscript.
- We have added an ethics statement plus further information on the consenting process.
- Apologies that you were unable to view Table 2. It is included at the end of the manuscript which has been reuploaded.
- We have updated the format of our manuscript to fit within the PLOS One guidelines which have been shared
- We have also updated our Supplementary Material to ‘Supporting Material’ and uploaded copies with and without tracked changes (‘SupportingInformation’ and ‘SupportingInformation with tracked changes’ respectively).
- Our updated conflict of interest statement is ‘MM is a scientific advisor to the UK Department of Health and Social Care and a paid consultant for Cepheid Inc (unrelated to COVID-19) who receives research funding from Roche (unrelated to COVID-19). This does not alter our adherence to PLOS ONE policies on sharing data and materials.

The remaining authors declare no other competing interests.

Please do not hesitate to contact us if you have any further questions or require clarification.

Yours sincerely,

Sara Graziadio, PhD

Senior Clinical Test Evaluation Methodologist, sara.graziadio@ncl.ac.uk

Joy Allen, PhD

Senior Clinical Test Evaluation Methodologist, joy.allen@ncl.ac.uk

On behalf of all the co-authors.
